# Climate as a Therapeutic Agent in Phthisis

**Published:** 1887-08

**Authors:** James Alexander Lindsay


### CLIMATE AS A THERAPEUTIC AGENT IN PHTHISIS.

I PROPOSE in this paper to consider four questions: 1. Does climate cure phthisis? 2. How does climate cure phthisis? 3. What climates cure phthisis? 4. What cases of phthisis are curable by climate?

1. Does climate cure phthisis? Yes, beyond question. To doubt this fact is to run counter to a vast mass of unassailable evidence and to give way to an unreasoning skepticism. I have met and talked with cured consumptives in many parts of the world—patients in whom the existence of the disease had been diagnosed by competent authorities, and the genuineness of whose recovery was testified by a long life of vigorous activity. These instances of complete recovery are, however, less common than cases of relative recovery, by which I mean those cases in which the morbid process in the lung becomes dormant and the patient lives out his days, but with diminished vitality, and maimed, as it were, for the rest of his life. It is an extremely important question to determine how many of these cases of cured phthisis are contingent upon the patient remaining in the country and climate where the cure was affected—are, in technical language, relative to his environment; and how many are so complete as to permit of a safe return to the unfavorable climatic conditions which prevail at home. Every practitioner who sees much chest disease has had distressing cases of consumptive patients who have gone abroad and apparently been cured, but who, yielding to that piteous home-sickness which sometimes assails the stoutest heart and disregards the plainest admonitions of prudence, have returned home, and returned to die. I fear that in a large proportion of cases the cure of phthisis is contingent and conditional, and I do not think we can often regard without serious concern the return of a cured consumptive to those climatic conditions which originally produced or predisposed to the disease.

2. How does climate cure phthisis? Not usually by a single or specific quality of the air or by any definite combination of meteorological conditions. The old notion of "healing air"—viz., that the air, by virtue of some inherent virtue, exercised a direct local curative influence upon tubercular deposits—is a delusion. Bournemouth and Arcachon profess to cure phthisis by the balsamic emanations from their pine forests, but if such emanations have any influence (which is quite doubtful), such influence is but a small and unimportant factor in their general climatic effect. The nearest approach to a specific climatic influence upon phthisis is found in the action of the climates of high altitudes, which has been abundantly proved to depend mainly upon the rarefaction of the air. To sum up this branch of the subject, climatic treatment cures phthisis by removing the consumptive from the evil influences of unfavorable meteorological conditions and of an injurious soil, and by transferring him to the climate where fresh air, sunshine, and an out-door life may be freely enjoyed, and where, in consequence, the processes of respiration, digestion, and sanguification proceed with sufficient energy to combat successfully the hereditary tendency or individual proclivity to pulmonary disease.

3. What climates cure phthisis? There are very many sanatoria which have been *alleged* to cure phthisis, but it is a significent circumstance that, while every year introduces some new claimant to attention, it also witnesses the dropping from the ranks of some discredited impostor. Time was when Montpellier was the reigning favorite. It is now almost forgotten. Pau and Nice have quite out-lived their once universal fame. Jersey, with its capital St. Heliers, once boasted great things, but we now know that its boasting was vain. The above losses of popularity have been amply justified, but other fluctuations of medical opinion and practice have been more arbitrary. The most favored health-resorts of the past generation were Madeira and Egypt. They are both at present under a cloud, but it will probably be a temporary cloud, as, for properly selected cases, these noted and long famous sanatoria have very decided advantages. The chief feature of modern practice is the great and growing popularity of the high altitude stations.

The number of health-resorts for phthisis is so vast that some classification must be attempted, though no satisfactory classification has yet been suggested. In default of better, let us divide sanatoria into three classes:—(*a*) The marine resorts. (*b*) The dry inland resorts. (*c*) The mountain resorts.

(*a*) The best marine resort, if I may so express it, is the ocean-going ship; on the high seas the peculiar features of the marine climate are enjoyed in perfection. Those features are great purity of the air and freedom from all sources of contamination, marked humidity, and very great equability, both as regards temperature and hygrometric condition. The general influence of ocean air is decidedly tonic, and improvement in appetite and gain in weight are almost universal on shipboard. The disadvantages of the sea voyage are — liability to sea-sickness, which is a serious difficulty in only 2 or 3 per cent of cases; the want of proper sleeping-room, and the consequent contamination of the air at night; and the absence of many home comforts. No consumptive should undertake a sea voyage unless he can afford to travel with tolerable comfort, pay for a roomy cabin, and have suitable companionship. The sailing-vessel should in nearly every case be preferred to a steamer, and the voyage to Australia or New Zealand *via* the Cape of Good Hope is the most adapted to the consumptive. The usual time of departure for Australia is September or October, but this is an unfortunate arrangement, as the invalid thus lands at the antipodes at the beginning of summer, the worst and most trying of the Australian seasons. A better plan is to set sail in July or August, so as to reach Australia during the charming and healthful time of spring. Sailing-vessels occupy from seventy to ninety days upon the passage. The sea voyage confers at least temporary benefit upon the great majority of consumptives, except those cases of great debility and prostration which should not be sent to sea. In the course of my four long voyages I have had as fellow-passengers a vast number of consumptives, who, with scarcely an exception, were happy and comfortable at sea, gained in weight, and experienced an amelioration of all their worst symptoms. It is a dictum of most writers upon the climatic treatment of phthisis that hæmorrhagic cases should not be sent to sea. I greatly doubt the accuracy of this view. I have only known two cases of hæmoptysis occurring at sea, and neither proved serious. I am quite unable to understand any theoretical ground on which hæmorrhage should be apprehended on shipboard. When the sea voyage fails, the causes of failure are often obvious. Excessive debility, rendering the patient unable to accommodate himself to the novel conditions of life at sea, sea-sickness, longing for home, which is apt to be strong amid the solitude of the ocean wastes, an uncomfortable ship, an over-crowded cabin, and an improper dietary —these often retard or prevent the beneficial influence of the sea voyage. There is one important point, of which my experience has afforded me several melancholy instances — viz., the proneness to relapse on reaching land. The patient should be warned to practice great caution as regards diet, exercise, and general habits on landing after a long voyage. Nearest to the climate of ocean comes that of ocean islands, such as Madeira, Teneriffe, the Azores, Nassau in the Bahamas, etc. Cases that have been known to do well on ship-board may be sent to these resorts, with tolerable confidence, the climatic conditions being similar. There is admirable accommodation at Madeira, and many cases do well there ; but diarrhœa is so epidemic as to be known as the " island complaint," and no case of phthisis should be sent there in which intestinal troubles have been much marked. The climate is very soft and soothing, and grateful to those suffering from bronchial or laryngeal complications, but it is deficient in tonic influence, and its effect upon the general course of the disease is decidedly inferior to that of more bracing localities. Patients may be sent there who lack the necessary vitality to enable them to react to tonic climatic conditions. Of the drier and more tonic marine resorts, Algiers, Tangier, and Malaga, are perhaps the best. The reputation of the Riviera is decidedly on the wane. Many seasons are delightful, but often the mistral is exceedingly treacherous, and snow-storms are not unknown. The chief stations in the Riviera are Hyères, Cannes, Nice, Monte Carlo, Mentone, Bordighera, and San Remo. Nice has quite lost its old reputation, owing to the sudden changes to which it is subject. Cannes is a lovely spot, but inferior to Mentone and San Remo for the purposes of the invalid. Where much shelter is a desideratum, Mentone should be preferred. Otherwise, San Remo possesses the best climate in the Riviera. Algiers is a great favorite at present. It possesses a dry, sunny, warm winter climate, with complete immunity from frost and fog. There are very few days on which the invalid cannot enjoy several hours of out-door life, and the proportion of charming weather is far higher than upon the northern shore of the Mediterranean. Consumptives should in no case reside in the town, which is very dusty and a prey to evil odors, but should chose the suburbs, such as Mustafa Inférieur or the Village d'Isly, and, as spring advances, a change inland to the dry and bracing climate of Hammam R'Ihra or Milianah may be advantageously adopted. Tangier resembles Algiers in its general climatic features, but the accommodation is inferior, and it is a very dull place. Malaga has a very good climate, but there is nothing to do, and patients soon weary of it. Biarritz is an excellent place in autumn, but too windy for a winter residence. Arcachon suits cases where nervous irritation is marked. As regards the marine resorts in Britian, Ventnor, Bournemouth, and Torquay are the best, and I have given them in what I consider as the probable order of merit. Ventnor is the warmest and driest of these resorts. Bournemouth has more shelter, and suits cases which cannot tolerate the more decided marine flavor of the air at Ventnor. Glengarriff is probably the best winter climate in Ireland. Rostrevor has some local repute, but it has little to recommend it. There are some excellent marine resorts in California, of which Santa Barbara is the best known. The Californian coast in its southern parts enjoys a remarkably dry, bright and tonic climate, well adapted for many cases of phthisis. Tasmania has a climate of great salubrity, but there are many very diverse climatic types included within the area of that small island. The west coast is stormy and wet, and wholly unsuitable to the invalid. The southern coast is somewhat too windy. The best regions for the consumptive are the north and northeast shores, which possess one of the brightest, mildest and most genial climates in the world. The coast region of Australia cannot in general be commended, being a sort of battle-ground for the breezes from the sea and the hot blasts from the interior, and the climate is in consequence fickle and stormy. The most favored region in Australia for the consumptive is the interior plain, of which I shall speak presently. The best marine resorts in New Zealand are Napier and Nelson. Speaking generally, the reputation of marine resorts has somewhat waned in proportion as the fame of the dry inland and the mountain sanatoria has increased. No marine resort is, I think, equal to the ocean voyage, where the latter can by enjoyed under the most favorable circumstances.

(*b*) The dry inland resorts. The best of these are Nubia, the interior parts of Algeria, the Orange Free State, and the vast interior plains of Australia, especially the Riverina of New South Wales and the Darling Downs of Queensland. I am inclined to rate very highly this type of climate in the treatment of phthisis. A large proportion of the cases of complete cure which I have known have been effected by a prolonged residence in some dry inland region, the cure, being, without doubt, in nearly every case materially promoted by the adoption of an open-air life, plain diet, and simple manners. Of the four regions mentioned, probably the Orange Free State is the best, as it possesses an advantage over the others in being at a considerable elevation. But personal considerations will lead in many cases to the selection of Australia, and it is no small advantage to send the consumptive to the land where he will meet his own kith and kin, where the language, food, and manners of the people will be familiar and congenial to him, and where he may possibly find such remunerative employment as may induce him to settle permanently. Speaking broadly, I would send to Australia only those consumptives to whom a dry inland climate would be thought to be beneficial, advising them in all cases to avoid the coast regions, and especially the capital towns, to go inland at once, settle upon some farm or sheep station, and make up their minds to a residence of at least two years. A large proportion of cases would thus be cured. To recommend Australia as a sanatorium for phthisis without keeping these points in view is to invite failure and disappointment.

(*c*) The mountain resorts. These sanatoria have sprung into sudden favor within the last twenty years, and their popularity grows yearly. The chief are Davos, Wiesen, St. Moritz, and the Maloja in the Alps; Colorado Springs and Manitou in the Rocky Mountains; Bogota, Jauja and Huancayo in the Andes The almost complete immunity from phthisis enjoyed by residents at high altitudes first suggested their adoption. The most important feature in the medical climatology of the mountain sanatoria is the rarefaction of the air, which promotes respiratory activity, the expansion of the chest, and the absorption of morbid deposits. The dryness, the purity of the air, and the freedom from organic dust are all of importance, but subsidiary to the atmospheric rarefaction. These sanatoria fall into two classes — the cold and the warm. In the Alps and Rocky mountains the winter climate is marked by perpetual snow and severe frost. In the equatorial Andes the climate at high altitudes is one of perpetual spring — humid, warm and vernal. The mountain resorts have proved most efficacious in the following cases: delayed recovery from pneumonia with threatening tuberculosis, chronic pleurisy with much of fibroid change, incipient catarrh of the apex, chronic tubercular phthisis, with good reaction and the retention of fair constitutional vigor, whether cases of primary disease limited in extent, or single cavity cases without tendency to extend. Hæmorrhagic cases do well, although the profession was long inclined to exclude such cases from the mountain treatment, owing to the unfortunate influence which a false analogical reasoning has exercised upon them. The contra-indications against the adoption of this line of treatment are: Weak circulation, which is absolutely prohibitory, senile change, the *eretische constitution*, extreme debility, and marked and progressive emaciation. Moderate pyrexia is not a hindrance, while sweating and fluxive diarrhœa are usually relieved at high altitudes. Where the cold mountain resorts fail the warm may often prove useful.

4. What cases of phthisis are curable by climate? I have hitherto spoken somewhat dogmatically, as the general principles of medical climatology and the general characters of the sanatoria for phthisis admit of very precise statement; but in dealing with this last question — to which I have more than once been obliged to make incidental allusion — I desire to speak with that diffidence which becomes the therapeutist. In many cases there are few more difficult problems than to determine whether a given case of phthisis will respond to climatic treatment, and what type of climate affords the best hope of success. Speaking generally, only chronic cases with fair reaction are suitable for climatic treatment. If the disease has a well-defined onset, and threatens to run an acute or semi-acute course; if the patient steadily loses ground and shows no gain in weight or other sign of rally under treatment; if the process in the lung is progressive and there are no evidences of repair — in each and all of these cases the interests of the patient will be best served by vetoing climatic treatment. The patient will die soon and die anywhere, and he may as well be allowed to pass his last days amidst the comforts and sympathies of home. Cases marked by circulatory weakness, with fast, feeble, fluttering pulse, slight cyanosis, and persistent coldness of the extremities, are very unfavorable for climatic treatment, and should on no account be sent to the mountains. Cases in which laryngeal or intestinal ulceration or renal complication have supervened upon the ordinary type of the disease should be allowed to die at home. Cases in which anæmia appears early and is well marked are unlikely to respond to climatic treatment. On the other hand, we see a vast number of cases of phthisis in which the onset is very gradual and the constitutional involvement for a long time slight. Such cases nearly always improve, even in this climate, under a system of high feeding, fresh air life, and cod-liver oil, and I entertain no doubt that a considerable proportion of them may be completely cured by removal to a suitable climate and the adoption of a prudent mode of life. In determining the climate to be chosen, it used to be taught that the condition of the bronchial mucous membrane was the chief guide: that cases with much bronchial catarrh should go to a dry climate, cases with dry irritable mucous membrane to a moist sedative climate, and so on. I greatly doubt the utility of this rule. Phthisis is not bronchitis, and all analogies for its treatment drawn from our knowledge of bronchitis are not merely unfruitful but misleading. In the therapeutics of consumption we have given up directing our medicinal treatment to the bronchial mucous membrane, and I am unable to see why we should still base our climatic treatment on a theory which we thus implicitly acknowledge to be unsound. Hippo, paregoric, and squills have almost disappeared from our treatment of phthisis; and climatic sanatoria, which soothes the patient's cough at the expense of his appetite and strength, must follow them into deserved oblivion. The consumptive does not die of his cough. He dies of progressive wasting. We have thrown aside expectorants and anodynes in favor of good food, exercise, and such aids to nutrition as cod-liver oil, hypophosphites, maltine, etc., and we must, when possible, choose climatic resorts which are tonic and stimulant rather than those that are mainly sedative. The vital point about any climate is, will it promote nutrition? In early uncomplicated cases with vigorous circulation I think the mountain climates offer the best hope. If the circulation be feeble, or if there be much nervous irritation, the choice will lie between the sea voyage and residence in such dry inland resorts as Upper Egypt or the interior plains of Australia. The sea voyage has the great recommendation that it rarely does harm, except in those very advanced cases which are beyond the reach of all treatment. If the patient objects to the mountains and shrinks from the long sea voyage, I think Algeria or Morocco should be preferred to France or Italy. Let me say, in conclusion, that climatic change is a snare instead of a help, a curse rather than a blessing, if it be regarded as a complete therapeusis in itself, and as enabling the patient to dispense with the usual lines of treatment and his customary precautions. Climate is not a specific. At best it is only a condition of cure, and we may expect it to be effectual only when the patient's food, habits, occupation, and mode of life are wisely regulated, so as to facilitate its beneficent influence. — *James Alexander Lindsay, A. M., M. D., in The Lancet.*

